# Presentation of a Complex Permittivity-Meter with Applications for Sensing the Moisture and Salinity of a Porous Media

**DOI:** 10.3390/s140915815

**Published:** 2014-08-26

**Authors:** Xavier Chavanne, Jean-Pierre Frangi

**Affiliations:** Institut de Physique du Globe de Paris, Univ. Paris Diderot, Sorbonne Paris Cité, UMR 7154 CNRS. Case postale 7011 - F75205 Paris cedex 13, France

**Keywords:** capacitive sensor, admittance measurement, impedance bridge, electrical circuit theory, calibration

## Abstract

This paper describes a sensor dedicated to measuring the vertical profile of the complex permittivity and the temperature of any medium in which sensor electrodes are inserted. Potential applications are the estimate of the humidity and salinity in a porous medium, such as a soil. It consists of vertically-stacked capacitors along two conductive parallel cylinders of 5 cm in diameter and at a 10-cm distance to scan a significant volume of the medium (∼1 L). It measures their admittances owing to a self-balanced impedance bridge operating at a frequency in the range of 1–20 MHz, possibly 30 MHz. Thanks to accurate design and electronic circuit theory-based modeling, the determination of the admittances takes into account all distortions due to lead and bridge electromagnetic effects inside the sensor when working at high frequencies. Calibration procedures and uncertainties are presented. The article also describes developments to make the present sensor autonomous on digital acquisition, basic data treatment and energy, as well as able to transfer stored data by a radio link. These steps in progress are prerequisites for a wireless network of sensors.

## 1. Introduction

Continuous, *in situ* and, potentially, real-time monitoring of moisture in a porous medium, like a soil, is in large demand in areas, such as agronomy, for the study of plants and their environment [1], civil engineering, to control ground stability under transport infrastructures or concrete hardening process, and hydrology, to better model the interactions between climate and soil moisture in a catchment area [2].

The most demanding applications necessitate measurements at different points over a large area, up to a few km^2^, with a resolution as high as 50 m [2]. They can be carried out every five minutes over a period of a few years. For each point, a moisture vertical profile is also required to determine water vertical flows, especially in arid climates. Such applications are met with a network of sensors automatically and remotely operated. Each one must be cost effective to deploy a large number of them without compromising on their robustness and accuracy. Such networks already exist using commercially available sensors [3].

The standard method—sample drying in oven—readily provides the mass of water content, but is too destructive and time consuming for continuous and *in situ* monitoring [4].

Among indirect methods, *i.e.*, relying on a physical property sensitive to the water content of a medium, techniques based on medium permittivity have been extensively used at least since 1980 [5].

The dielectric permittivity *ε* of a material represents the coefficient of proportionality in the constitutive relation between its electric field E and its electric displacement D (Section 1.4 of [6]). E includes the effects of electric charges and material polarization due to its dipoles, while the flux of D over a surface results only from free charges, including those on conductors inside or close to the material, in the volume enclosed by the surface. In absence of a free charge in the material, the value of *e* normalized by vacuum permittivity *ε*_0_ (*ε*_0_ = 8.854 pF·m^−1^), or relative permittivity *ε_r_*, is about *ε_r_* = 80 for pure water, whereas it amounts to about *ε_r_* = 4 for solid constituents of soil and *ε_r_* = 1 for air. These values explain the method's interest.

Free charges, when present, produce a current in response to the electric field. According to Ohm's law of diffusive transport, current is proportional to E with the coefficient given by conductivity *σ*. When using alternating fields at a frequency *f*, both dipole polarization and conduction by charges are taken into account, owing to the complex dielectric permittivity *ε*:

(1) $$\varepsilon =\varepsilon_{0}\varepsilon_{r}-\text{j}\frac{\sigma }{\omega }$$

where **j**^2^ = − 1 and *ω* is the angular frequency *ω* = 2* π f*. In the article, relative permittivity *ε_r_* corresponds thus to the real part of the complex permittivity normalized by the permittivity in a vacuum.

In the case of heterogeneous media, such as soils, and because of the large size of sensing instruments relative to medium pores, *ε_r_* and *σ* represent averaged or apparent quantities. However, they remain soil specific and should not depend on sensor geometry. It requires a large spatial distribution of the electrical field applied by the sensor in the medium, preferably over a volume of 1 dm^3^ or a size *D* of 10 cm. A large volume around the sensor also reduces the part of the medium modified by sensor insertion. The volume depends on both the distance between sensor electrodes and their diameters.

Due to the heterogeneity of a porous medium and the presence of water with ions, average medium polarization results in part from the existence of dipoles at the scale of the pore [1]. When f is increased, relaxation of the polarization due to thermal diffusion arises, modifying the values of apparent *ε_r_* and *σ* (the Maxwell-Wagner relaxation). The effect of the relaxation for one dipole is well described by the Debye function. *ε_r_* decreases from a constant value at frequencies lower than the relaxation frequency *f_r_* to another constant at higher frequencies. *σ* presents a maximum around *f_r_* due to relaxation loss. For a complex medium, such as a soil, due to the various mechanisms of polarization, variation of *ε_r_* and *σ* with frequency presents a much broader band. Over this range, *ε_r_* depends not only on the water content of the medium, but also on parameters, such as its texture, mineralogical composition and salinity. To reduce their influence, permittivity meters have to work preferably above this relaxation domain. Laboratory measurements using sands with an amount of clay lower than 10% (on a dry basis) show the end of Maxwell-Wagner relaxation below 10 MHz [7-10]. This limit can be exceeded in the case of a large content of water at high salinity; typically higher than 30% with water salinity above 0.1 S/m [10]. Contents of kaolite or bentonite clays at 20% and higher seem to shift the relaxation of polarization toward a lower frequency than in sands at same conditions, while increasing the final value of *ε_r_* at 10 MHz. On the other hand, permittivity measurements using pure kaolite and bentonite with a mass of water one- to six-times as high as the mass of the dry clays indicate that the maximum of clay polarization is at a frequency lower than 100 Hz. This progressively decreases to a limit just above 1 MHz (with the frequency of measurements limited at 13 MHz) [11].

On the other hand, at much higher frequencies, molecular dipoles start relaxing. *f_r_* is close to *f_r_* = 10 GHz for dipoles of unbound molecules of water. For molecules bound to the surface of clay or other particles in soil, relaxation occurs at lower *f_r_* than for free molecules, down to 10 kHz, according to Hilhorst [1]. Because they represent one layer of molecules, their contribution is assumed to be negligible.

Values of *ε_r_* expected for soils at 20 MHz range from 2 to 50, while those for *σ* range from nearly zero to 0.1 S-m^−1^ [1].

Besides the complex interpretation of a porous medium as a dielectric, a difficulty arises from the capability of instruments to determine accurately the apparent *ε_r_* and *σ* of the medium.

Instruments to estimate *ε_r_* or *ε* produce in the medium under study electromagnetic fields propagating along a direction, x, which are solutions of Maxwell's electromagnetic equations [6] (Introduction). They present the general form of *A*(**r**) *ph*(*x*/*λ* − *ωt*), where *A* is the field amplitude at point r in the medium, *ph* the field phase and λ their electromagnetic wave length. Both *A* and λ depend on *ε*, which permits its measurement. The expression of λ is:

(2) $$\lambda =\frac{c}{\omega \text{Re}(\sqrt{\varepsilon /\varepsilon_{0}})}\approx \frac{c}{\omega (\sqrt{\varepsilon_{r}})}$$

where *c* is the velocity of electromagnetic waves in a vacuum (*c* approximately 0.30 Gm·s^−1^). *Re*(*X*) is the real part of the complex *X*. *ε* is given by Equation (1).

The wavelength determines two types of instruments.

For λ lower than the size *D* ∼ 10 cm of the medium scanned by sensor fields, field propagation is the dominant phenomenon. The sensor operating frequency varies from about 100 MHz to 3 GHz. An estimate of *ε_r_* is usually based on the measurement of λ or phase velocity *ω* λ in the medium, according to Equation (2). Examples of such sensors are ground penetrating radars using free pulses, time domain reflectometry (TDR) or transmission (TDT) probes using step waves propagating along metallic rods [5,12] and phase transmission probes (VIRRIB) using sinusoidal waves along two concentric poles. $\text{Re}(\sqrt{\varepsilon /\varepsilon_{0}})$, and so, the estimate of *ε_r_* are deduced from the round-trip time or phase shift of waves along the field path in the medium. Values can be impacted by *σ* in soils with great salinity. A means to overcome this difficulty is to measure the attenuation of waves along the distance back to the sensor, which is related to the imaginary part of $\sqrt{\varepsilon /\varepsilon_{0}}$. Distortions of acquired waveforms and multiple reflections can reduce the accuracy to measure round-trip time and attenuation, especially the latter.

For λ larger than *D* ∼ 10, which means an operating frequency *f* below or close to 100 MHz, but still larger than 10 MHz, electromagnetic propagation becomes negligible. The field form is approximately *A*(**r**)*ph*(*ωt*). Electromagnetic phenomena are then modeled by the electric circuit theory (Section 5.18 of [6]). Fields and vectors are converted into voltages and currents. The system made of sensor electrodes and the medium is modeled by its capacitance or impedance. Capacitance is directly related to *ε* (Equation (1)).

Currently available capacitance sensors actually measure either the frequency of an electronic oscillator comprising a capacitor (e.g., the EnviroSCAN probe marketed by Sentek Pty Ltd. [13]) or the root mean square of charging and discharging cycles of a capacitor (the series of probes commercialized by Decagon Devices [14]). In both cases, only a part of the capacitor dielectric is made of the medium to be analyzed. Besides, conversion of capacitance to permittivity is affected by a field fringing effect. Hence, the sensor output is only related to *ε_r_* thanks to semi- or totally empirical relations [15]. Manufacturers often prefer to provide a direct calibration of the sensor signal against the water content of the medium. However, because the relation depends not only on medium parameters, but also on the sensor geometry and electronic circuit, especially at a frequency above 10 MHz, the relation is not universal. Moreover, the dependence of the sensor output on circuit resistance (hence, on the *σ* of the medium) due to the technique of measurement itself can make the permittivity determination incorrect [13,14,16]. Some devices, like part of the Decagon sensors, perform *σ* reading to correct the signal for its effect. However, measurements are not carried out in the same conditions of geometry and frequency as those for *ε_r_*.

Other sensors using the impedance measurement technique operate with sinusoidal waves propagating in an open-ended transmission line, usually a coaxial one [8,17]. The cable is terminated by a probe inserted into the medium. From the complex coefficient of wave reflexion at the cable end, these can deduce the impedance of the probe. A relation in complex notation exists between the impedance and medium permittivity, usually only valid for λ lower than the probe length (e.g., 6 cm at 50 MHz [8]). This device is often used in conjunction with a network analyzer to derive dielectric permittivity spectra through frequency scanning. These correspond to laboratory equipment and operate with small or homogeneous samples at a frequency above 100 MHz (e.g., Agilent 8753D equipped with probe Agilent 85070E)

As a result of the difficulties in directly obtaining the permittivity, it is not uncommon to observe a dispersion of values of *ε_r_* among different sensors for a given soil and its water content, even for sands [2].

The sensor described in the present article pertains to the capacitance category. Its principle is based on the direct measurement of the complex admittance *Y* of sensor electrodes imbedded in the medium under study. As a result of the dipoles and charges in the medium, *Y* is equivalent to a capacitor in parallel with a conductor:

(3) Y=G+jCω

*Y* is thus directly related to the apparent permittivity of the medium *ε* in Equation (1) according to:

(4) Y=jεωg,

where *g* is a length depending only on the geometry of electrodes.

To our knowledge, only a few examples of such a type used for soil or other porous media exist [1,18]. They usually consist of one capacitor for which the size order is 1 cm, *i.e.*, scanning a small medium volume.

A previous prototype developed by our group deduces *Y* from the alternating voltage applied between the electrodes and the resulting current owing to Ohm's law [19,20]. The present article introduces another version using a self-balanced impedance bridge. Both are part of the sensor described in the patent Frangi *et al.* [21] with the bridge itself presented in the patent Galaud and Bruère [22]. The bridge output corresponds to two constant voltages related, respectively, to *G* and *C*. This permits one to achieve the goal of an autonomous sensor using simple electronics to acquire and store signals. The accuracy is improved, as well, compared to the previous technique. On the other hand, the bridge operates only at one frequency and, thus, does not allow the easy study of the variation of the *ε* of the medium with *f*.

The determination of the admittance *Y* requires the correction of all electromagnetic perturbations introduced by leads and electronic circuits inside the sensor. They are all the more important since the operating frequency is high (here, *f* = 20 MHz) and the dimensions large (*D* ∼ 10). This step is necessary in order to obtain dielectric parameters specific to the porous medium under study, which can be easily interpreted by dielectric models of the medium.

The first section describes the geometry and the electronic layout of the sensor. In particular, it operates at 20 MHz with multiple channels to trace a vertical profile of *ε*. The section also presents a circuit theory-based model to determine *Y* from sensor outputs taking into account all the parasitic effects of the sensor circuit.

The calibration procedures based on the model and resulting uncertainties on *Y* are presented in the second section.

The third section presents ongoing developments to make the sensor autonomous on digital acquisition, basic data treatment and storage. It will also be able to transfer its data by a radio link towards a central station for further analysis. These features are prerequisites for a wireless network of sensors.

## 2. Main Characteristics of the Sensor

### 2.1. Geometry and Electronic Layout

**Figure 1.**
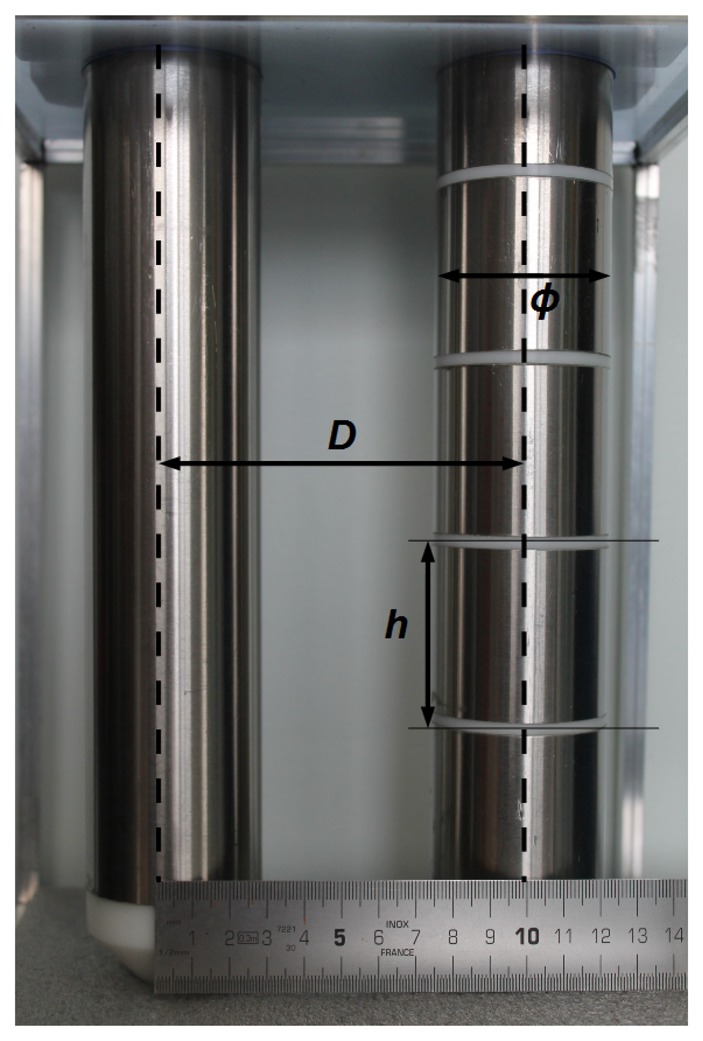
Photograph of sensor electrodes and their five channels. The dimensions for one channel are shown by a ruler: *ϕ* is the electrode diameter (*ϕ* = 50 mm), *D* the distance between their axes (*D* = 100 mm) and *h* their height (*h* = 50 mm).

In order to obtain a vertical profile of the permittivity of the medium *ε*, the sensing part of the present probe consists of five stacked capacitors, or channels, along two parallel cylinders (Figure 1). The two cylinders are made of stainless steel tubes to constitute the channel electrodes. The height and diameter of one channel electrode is 50 mm. Steel tubes are placed around and supported by a tube of polyoxymethylene (POM or Delrin, its trademark). For one of the cylinders, the plastic frame also provides a 5-mm insulating ring between channels. The other cylinder is made continuous over the channels, as it does not need this separation, and its manufacturing is thus simpler.

The sensor's overall height and the number of channels can be increased for deeper study of the soil.

Except for the two end capacitors, or guards, which concentrate the fringing effects, the electrical field of the capacitors resulting from a voltage applied between their two electrodes lies ideally in the planeperpendicular to the electrodes. For a capacitor made of two parallel electrodes of height *h*, diameter *ϕ* and axes distant of *D* (see Figure 1), the factor *g* in Equation (4) is [23]:

(5) $$g=\frac{h\pi }{\text{arccosh}(\text{D}/\phi )}.$$

The value of *g* for the present sensor is 0.12 m (*h* = 50 mm, *D* = 100 mm and *ϕ* = 50 mm).

**Figure 2.**
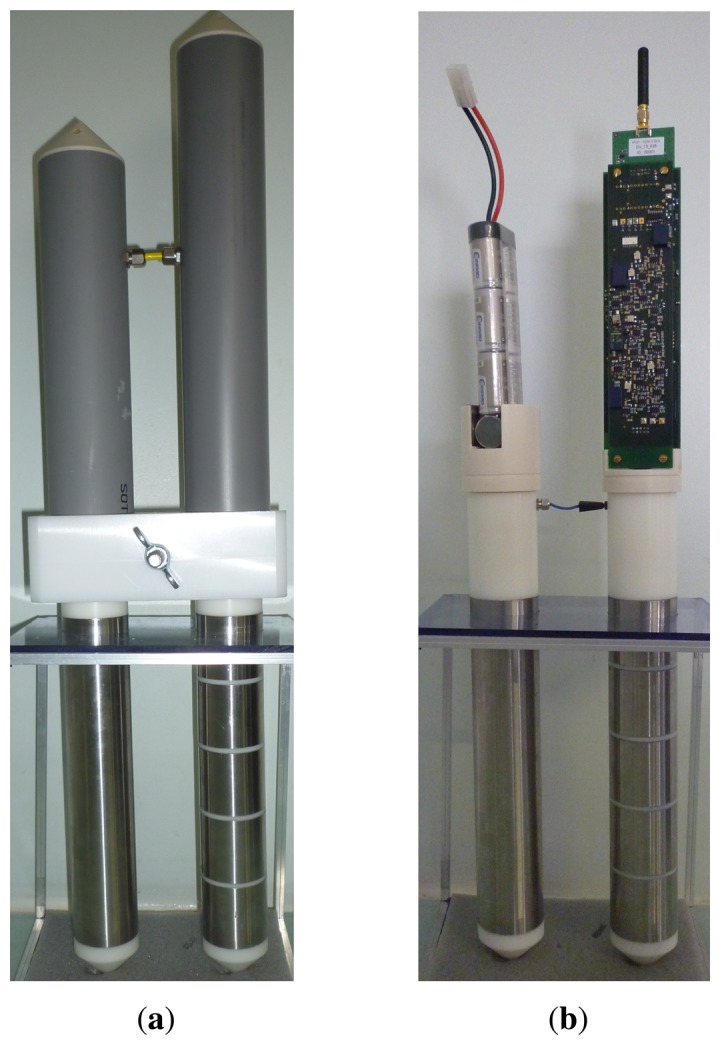
Photograph of the whole sensor with its electrodes and two aerial polyvinyl chloride tubes. Tubes house, respectively, the printed circuit boards and the battery for autonomous operations. (**a**) With the tubes; (**b**) Without the tubes.

The channel electrodes are wired to a printed circuit board located above the discontinuous cylinder and housed in a polyvinyl chloride tube (Figure 2). The circuit contains a self-balanced impedance bridge to measure the admittance *Y_x_* of each channel at a fixed frequency *f* (the present prototype operates at *f* = 20 MHz). The bridge itself is manufactured by the company CAPAAB (Chatenay Malabry France). Remotely operated relays allow one to switch successively each channel to the bridge.

The bridge applies an oscillating voltage *v_osc_* between sensor electrodes to generate an alternating current *i_x_* through *Y_x_* of each channel. When the channel is connected to the bridge input, *i_x_* is added to a current *i_eq_* produced by the bridge. The sum is sent to the inverse input of a trans-impedance, which is set at the bridge ground to maintain the voltage *v_osc_* between electrodes. Owing to a control loop made of multipliers for demodulation, modulation and integrators, the bridge automatically adjusts the current *i_eq_* to cancel out the sum *i_x_* + *i_eq_*. To achieve this operation, the feedback produces a constant voltage *V*_*G*_ to adjust the part of *i_eq_* in phase with *v_osc_* and a voltage *V_C_* to adjust the part of *i_eq_* in quadrature of phase with *v_osc_*. Both voltages constitute the sensor output and are related to the conductance *G_x_* and the capacitance *C_x_* of *Y_x_*, respectively. Their values range from −4 V to 4 V. A negative admittance for the imaginary part means an inductance effect.

A measurement is achieved with no channel connected to the bridge in order to determine the bridge residual output, typically less than a few tens of mV. This residual is removed from the raw output of each channel to improve the linearity between the resulting sensor signals, Δ*V_G_* and Δ*V_C_*, and *Y_x_*. The operation is carried out at each set of measurements as the residual varies with time.

Thereafter, the chain of measurement is modeled using Δ*V_G_* and Δ*V_C_*, even for the very first operations that handle the raw *V_G_* and *V_C_*. Their modeling does not depend actually on the choice of voltages.

The printed board also comprises a reference channel made of a known capacitor and resistor. This allows for controlling any drift of the coefficients of conversion between *Y* and its output Δ*V_Y_* due to the effects of aging and temperature.

### 2.2. Electronic Modeling

#### 2.2.1. Ideal Sensor

**Figure 3.**
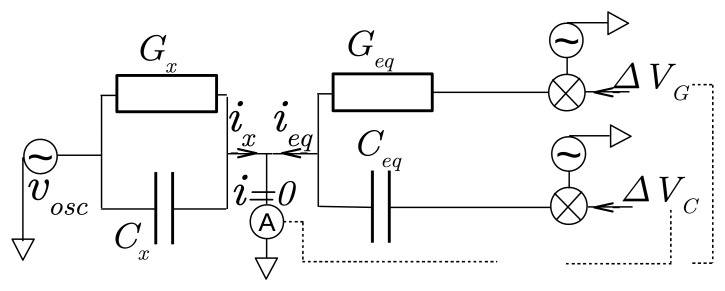
Schematic view of an ideal admittance bridge. When in balance, the current *i_eq_* generated by the bridge through the conductance *G_eq_* under the voltage *v_osc_* Δ*V_G_* (using a multiplier) and through the capacitance *C_eq_ω* under the voltage *v_osc_* Δ*V_C_* offsets the current *i_x_* from the admittance to be determined, *Y_x_* = *G_x_* + **j** *C_x_ω. ω* is the bridge angular frequency in rad·s^−1^.

The principle of the admittance bridge is summarized in Figure 3. Thanks to multipliers, the bridge produces two alternating voltages, which are the product of *v_osc_* with Δ*V_G_* or Δ*V_C_*. They generate the current *i_eq_* through fixed admittances *G_eq_* and *C_eq_ω*. When the bridge is in balance, *i_eq_* offsets exactly the current *i_x_*. Assuming ideal electronic operations, we have then:

(6) $$i_{eq}=G_{eq}v_{\text{osc}}\Delta V_{G}+\text{j}C_{eq}\omega v_{\text{osc}}\Delta V_{C}$$

The coefficients *G_eq_* and *C_eq_ ω* are determined by the choice of electronic components in the bridge circuit. They can be thus made negative and equal for the ease of data treatment.

Hence, for an ideal bridge, Δ*V_G_* and Δ*V_C_* are related to *Y_x_* according to:

(7) $$\{\begin{array}{l} G_{x}=-G_{eq}\Delta V_{G}\\ C_{x}\omega =-C_{eq}\omega \Delta V_{C} \end{array}$$

A typical value for *G_eq_* and *C_eq_ ω* is 3.0 *μ*S·mV^−1^. Given the range of Δ*V_G_* and Δ*V_C_*, as well as Equations (1), (4) and (5), the sensor specifications permit one to cover the range 0-0.1 S·m^−1^ for *σ* (or 0-12 mS for *G*) and the range 1-100 for *ε_r_* (approximately that for *C* in pF).

However, interferences between channels, bridge imperfections and parasitic impedances of channel leads introduce second order parameters in Equation (7). They are all the more important since the frequency *f* is high and the sensor size large. In order to have a comprehensive understanding of them and to reduce the number of their parameters, their modeling is performed using the electric circuit theory.

#### 2.2.2. Diaphony

Actual outputs $\Delta V_{Gk}^{\prime }$ and $\Delta V_{Ck}^{\prime }$ for channel *k* are distorted by the outputs from channel *m*, $\Delta V_{Gm}^{\prime }$ and $\Delta V_{Cm}^{\prime }$. Disturbances between the two channels, or their diaphony, are of two types. The first one results from the influences between channel leads before relay inputs, chiefly through mutual inductance. The second type results from the interferences occurring in the printed circuit board.

**Figure 4.**
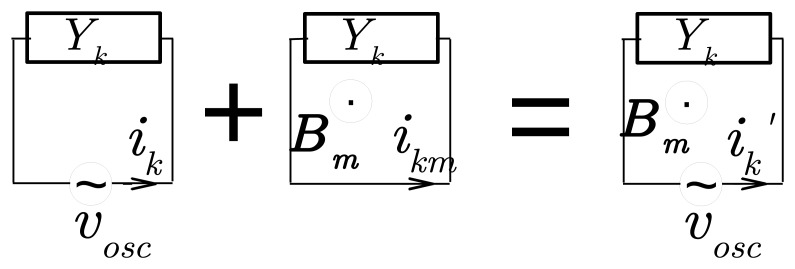
The total current $i_{k}^{\prime }$ in the circuit of channel *k* comprises a contribution *i_k_* due directly to the admittance *Y_k_* under study and another *i_km_* due to the influence of channel *m* through its magnetic field *B*_m_.

Let us examine the first type by modeling it according to the electromagnetic laws on mutual inductance (Section 5.17 of [6]). The current $i_{m}^{\prime }$ in channel *m* generates a magnetic field *B*_m_. In turn, it produces via its flux *ϕ_km_* through the circuit of channel *k* an additional current *i_km_* in *k* (Figure 4):

(8) $$\begin{array}{ll} i_{k}^{\prime } & =i_{k}+i_{km}\\ & =Y_{k}v_{\text{osc}}+Y_{k}\left[-\frac{d\Phi_{km}}{dt}\right] \end{array}$$

where the magnetic flux Φ*_km_* is expressed by:

(9) $$\Phi_{km}=M_{km}i_{m}^{\prime }$$

The factor *M_km_* depends on the geometry of circuits *k* and *m* and the distance between them.

The currents $i_{k}^{\prime }$ and $i_{m}^{\prime }$ sent to the bridge correspond respectively to admittances $Y_{k}^{\prime }$ and $Y_{m}^{\prime }$, which include the mutual inductance effect. Equation (8) becomes:

(10) $$Y_{k}^{\prime }=Y_{k}-\text{j}\omega M_{km}Y_{k}Y_{m}^{\prime }≃Y_{k}-\text{j}\omega M_{km}Y_{k}^{\prime }Y_{m}^{\prime }$$

Decomposing into real and imaginary parts, we have:

(11) $$\{\begin{array}{l} G_{k}^{\prime }≃G_{k}+\omega M_{km}(G_{k}^{\prime }C_{m}^{\prime }\omega +C_{k}^{\prime }\omega G_{m}^{\prime })\\ C_{k}^{\prime }\omega ≃C_{k}\omega +\omega M_{km}(C_{k}^{\prime }C_{m}^{\prime }\omega^{2}-G_{k}^{\prime }G_{m}^{\prime }) \end{array}$$

Converting the admittance parts into bridge output according to Equation (7) applied to each channel:

(12) $$\{\begin{array}{c} \Delta V_{Gk}^{\prime }≃\Delta V_{Gk}+\omega M_{km}C_{eq_{m}}\omega \Delta V_{Gk}^{\prime }\Delta V_{Cm}^{\prime }+\omega M_{km}\frac{C_{eq_{k}}\omega }{G_{eq_{k}}}G_{eq_{m}}\Delta V_{Ck}^{\prime }\Delta V_{Gm}^{\prime }\\ \Delta V_{Ck}^{\prime }≃\Delta V_{Ck}+\omega M_{km}C_{eq_{m}}\omega \Delta V_{Ck}^{\prime }\Delta V_{Cm}^{\prime }+\omega M_{km}\frac{G_{eq_{k}}}{C_{eq_{k}}\omega }G_{eq_{m}}\Delta V_{Gk}^{\prime }\Delta V_{Gm}^{\prime } \end{array}$$

When *G_eq_k__* is equal to *C_eq_k__ω*, which is then valid for all channels, the coefficients in the last two terms of the right-hand side of the equations are identical.

The inductance theory does not readily provide the coefficients *M_km_*. A calibration in which the output of both channels *k* and *m* are changed independently over a large range is required.

Moreover, the calibration shows the necessity to introduce on $\Delta V_{Yk}^{\prime }$ another contribution from channel *m* in the form *a_km_* $\Delta V_{Ym}^{\prime }$. This represents the second type of interferences. It does not appear symmetrical between two channels, like in the case of the mutual inductance. Neither is it identical between branches *Yk* ≡ *Gk* and *Yk* ≡ *Ckω*, which necessitates distinct coefficients, *ag_km_* and *ac_km_*.

As a result, the output Δ*V_Gk_* and Δ*V_Ck_* resulting only from the admittance *Y_k_* in channel *k* is obtained by the correction of *k* actual output from the distortions due to all other channels according to:

(13) $$\{\begin{array}{c} \Delta V_{Gk}≃\Delta V_{Gk}^{\prime }-\sum_{m\neq k}\left[(ag_{km}+f_{km}\Delta V_{Ck}^{\prime })\Delta V_{Gm}^{\prime }+f_{km}\Delta V_{Gk}^{\prime }\Delta V_{Cm}^{\prime }\right]\\ \Delta V_{Ck}≃\Delta V_{Ck}^{\prime }-\sum_{m\neq k}\left[-f_{km}\Delta V_{Gk}^{\prime }\Delta V_{Gm}^{\prime }+(ac_{km}+f_{km}\Delta V_{Ck}^{\prime })\Delta V_{Cm}^{\prime }\right] \end{array}$$

Each type of coefficient, *ag_km_*, *ac_km_* and *f_km_*, is expressed in the form of a matrix of which rows correspond to measuring channels, while columns correspond to all channels, including the upper and lower guards. Values for *ag_km_* and *ac_km_* are 0.02-0.01 at most, while *f_km_* varies from zero to 10^−5^ mV^−1^.

#### 2.2.3. Bridge Imperfections

**Figure 5.**
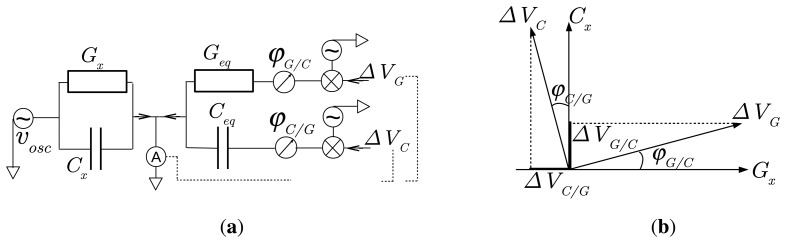
Bridge phase drifts *φ_C/G_* and *φ_G/C_*. (**a**) Schematic view of the admittance bridge as in Figure 3 taking into account the phase drifts *φ_G/C_* and *φ_C/G_* due to multipliers at the conductance and capacitance branches, respectively; (**b**) Voltage interferences Δ*V_C/G_* and Δ*V_G/C_* between the conductance and capacitance branches, due to the phase drifts *φ_C/G_* and *φ_G/C_*.

Even after correcting for its diaphony effect, the bridge output does not match exactly Equation (7). Multipliers in the bridge to obtain the products *v_osc_* Δ*V_G_* and *v_osc_* Δ*V_C_* introduce the phase drifts *φ_G/C_* and *φ_C/G_*, respectively (Figure 5a). As a result, the output Δ*V_G_* of the conductance branch presents a contribution Δ*V_C/G_* from the output Δ*V_C_* of the capacitance branch, and *vice versa*. From vector projections in Figure 5b, Equation (7) becomes:

(14) $$\{\begin{array}{l} -G_{x}=G_{eq}\Delta V_{G}\text{cos}(\varphi_{G/C})-C_{eq}\omega \Delta V_{C}\text{sin}(\varphi_{C/G})\\ -C_{x}\omega =C_{eq}\omega \Delta V_{C}\text{cos}(\varphi_{C/G})+G_{eq}\Delta V_{G}\text{sin}(\varphi_{G/C}) \end{array}$$

#### 2.2.4. Channel Parasitic Impedances

**Figure 6.**
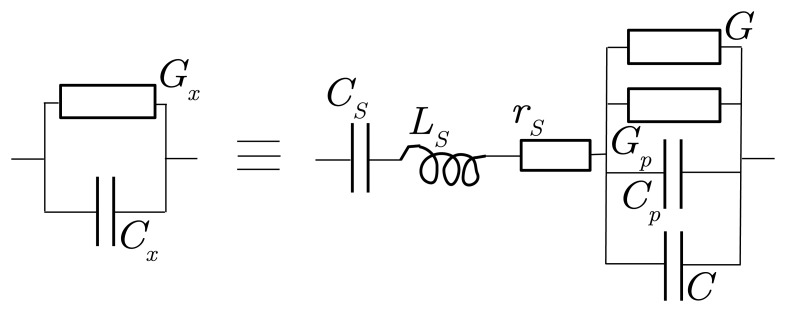
The breakdown of the admittance *Y_x_* = *G_x_* + **j***C_x_ω* actually measured by the bridge. In addition to the admittance *Y* = *G* + **j** *Cω* under study, *Y_x_* includes a capacitance *C_S_*, an inductance *L_S_* and a resistance *r_S_* in series, as well as parasitic admittances in parallel of *Y*, *Y_p_* = *G_p_* + **j** *C_p_ω*, all due to the leads.

The admittance *Y_x_* measured by the bridge and obtained from Equation (14) comprises not only the admittance *Y* specific to the medium under study, but also those of the connection between electrodes and bridge inputs (Figure 6). They are equivalent to parasitic impedances in series—a capacitance *C_S_*, an inductance *L_S_* and a resistance *r_S_*—and an admittance in parallel: *Y_p_* = *G_p_* + **j** *C_p_ω*.

Hence, the expression of *Y_x_* is:

(15) $$Y_{x}=\frac{Y+Y_{p}}{1+Z_{S}(Y+Y_{p})},$$

with

$$Z_{S}=r_{S}+\text{j}L_{S}\omega +\frac{1}{\text{j}C_{S}\omega }=r_{S}+\text{j}L_{S}\omega (1-\frac{1}{L_{S}C_{S}\omega^{2}})=r_{S}+\text{j}L_{S}^{\prime }\omega .$$

The parallel conductance *G_p_* is typically 10-40 *μ*S, while the capacitance *C_p_* resulting from the proximity of the lead carrying the potential *v_osc_* to the one for ground is about 0.5 pF.

The resistance *r_S_* is about a few ohms at *f* = 20 MHz, higher than at a low frequency, due the current skin effect in leads (Section 5.18.1 of [6]).

A straight wire of one meter length presents a typical self-inductance of 1 *μ*H. One meter is close to circuit length of the channel farthest from the bridge board (the lower guard). The circuit inductance *L_S_* results in an impedance as large as 120 Ω at *f* = 20 MHz. Owing to the addition of a capacitor *C_S_* in series, *L_S_* is mostly canceled. The resulting inductance $L_{S}^{\prime }$ in *Z_S_* of Equation (15) is thus much smaller. This adjustment is possible as long as the bridge operates at a fixed angular frequency *ω*.

#### 2.2.5. Summary of Required Coefficients of Conversion

Raw output $\Delta V_{Gk}^{\prime }$ and $\Delta V_{Ck}^{\prime }$ for the channel *k* is first corrected from the diaphony due to the sensor's other channels *m* according to Equation (13). The operation requires three matrices of coefficients, of which the size depends on the total number of channels and the number of measuring channels. Accounting for three channels of interest and five channels overall, the procedure must be applied to 12 pairs of channels.

Subsequently, for each measuring channel, a set of eight coefficients are necessary to convert the diaphony free voltages Δ*V_Gk_* and Δ*V_Ck_* into the admittance *Y* of the medium:

- The first order coefficient *G_eq_* and *C_eq_ω* in *μ*S mV^−1^ (Equation (7)).
- The bridge phase drifts *φ_C/G_* and *φ_G/C_* in rad (Equation (14)).
- The parasitic admittances—*G_p_* and *C_p_ω* in *μ*S—and impedances— $L_{S}^{\prime }\omega$ and *r_S_* in Ω (Equation (15)).

The two last types of coefficients correspond to second order parameters resulting from identified and unavoidable phenomena.

It should be noted that sensors with identical characteristics of leads, printed board and its components (especially those controlling the above parameters) will present the same diaphony and direct coefficients within an interval of less 1% (reproducible values according to specifications of the components from the manufacturers). Hence, calibration can be carried out for a sample of a few sensors among a large number.

The only exception is the two bridge phase drifts, which can vary from one bridge to another.

## 3. Calibration and Measurement Uncertainty

For the ease of operations, calibration is performed with reference capacitors *C_ref_* and resistors *R_ref_* as done in a previous study [19]. A calibration with pure liquids of known properties has shown good agreement with that using electronic components, taking into account all source of errors [20].

*C_ref_* and *R_ref_* are chosen to present the lowest parasitic impedances at *f* = 20 MHz. Capacitors present a parallel conductance lower than 0.5 *μ*S. Resistor stray capacitance is determined by the bridge itself due to its high resolution. It amounts to 0.10 pF. The U1733C LCR meter of Agilent operating at *f* = 100 kHz permits one to measure the capacitance of the references within the uncertainty interval of +/− 0.01 +/− 0.2% *C* pF.

The first step consists in reducing mechanically the main second order parameters, namely the two phase drifts of the bridge and the self-inductance of channel leads.

### 3.1. Calibration Procedure

#### 3.1.1. Mechanical Adjustments

This step requires access to the circuit of the bridge on the printed board.

Each branch of the bridge disposes of an electronic circuit with an adjustable potentiometer to reduce the phase drift introduced by the branch multiplier (Figure 5a). A few capacitors for the conductance branch or a few resistors for the capacitance one are successively placed at the bridge inputs while measuring the direct output voltage of the branch. The potentiometer is adjusted in order to make the branch output nearly non-sensitive to any admittance in the quadrature of phase with the branch normal phase.

The procedure takes place with all relays in open mode to disconnect the bridge from the channels (or before connecting them to the printed circuit board).

The next step is the insertion of an offsetting capacitor in the circuit of each channel before the relay (see Section 2.2.4). Its capacitance *C_S_* is fixed according to Equation (15) with a value of *L_S_* determined by a first calibration using *C_ref_*. To make the diaphony due to other channels small enough, no component is attached at their electrodes, and *C_ref_* is larger than 8 pF.

#### 3.1.2. Diaphony Calibration

The rest of the procedure is performed with the sensor in the same conditions as for a field trial, *i.e.*, the same chain of measurement and the polyvinyl chloride tubes put in place.

For each pair *k* and *m*, their output voltages $\Delta V_{Yk}^{\prime }$ and $\Delta V_{Ym}^{\prime }$ are varied independently (*Y* is either *G* or *C*). In practice, for a fixed admittance in the channel *k* (resistance or capacitance) a set of three capacitors and three resistors are placed in *m*. Another admittance is attached in *k*, and the same set of admittances is again used in *m*. For a complete and symmetrical study of the pair of channels, the same set of admittances is used in *k* as in *m*.

By applying linear regressions at selected sets of data, such as $\Delta V_{Gk}^{\prime }$ in function of $\Delta V_{Gm}^{\prime }$ at nearly constant $\Delta V_{Ck}^{\prime }$, the various coefficients—*ag_km_*, *ac_km_* and *f_km_*—are determined.

They are further adjusted by minimizing the variation of the output of channel *k* corrected according to Equation (13), when the admittance on the channel *k* is fixed and the admittance on the channel *m* is changed.

#### 3.1.3. Channel Calibration

This part of the procedure is carried out with the channel output Δ*V_G_* and Δ*V_C_* corrected from the diaphony using the parameters determined previously. Moreover, other channels have no admittance, except that due to air. Consequently, the diaphony contribution is negligible.

Assuming now with good approximation small values for *Z_S_* and *Y_p_*, Equation (15) is developed in power of *Y*, retaining only the first order contributions from *Z_S_* and *Y_p_*. It permits one to determine more easily the coefficients of one channel from its calibration. Hence, voltages Δ*V_G_* and Δ*V_C_* in Equation (14) are expressed as a function of *Y* according to a second order polynomial law:

(16) $$\{\begin{array}{l} -(G+G_{p})(1-r_{S}G+2L_{S}^{\prime }C\omega^{2})-{\left(C\omega \right)}^{2}r_{S}≃G_{eq}\Delta V_{G}-C_{eq}\omega \Delta V_{C}\varphi_{C/G}\\ -(C+C_{p})\omega (1-2r_{S}G+L_{S}^{\prime }C\omega^{2})+G^{2}L_{S}^{\prime }\omega ≃C_{eq}\omega \Delta V_{C}+G_{eq}\Delta V_{G}\varphi_{G/C} \end{array}$$

When using reference capacitors *Y* ≡ *C_ref_ ω*, we have from Equation (16):

(17) $$\{\begin{array}{l} -G_{p}-C_{\text{ref}}\omega \varphi_{C/G}-{\left(C_{\text{ref}}\omega \right)}^{2}r_{S}≃G_{eq}\Delta V_{G}\\ -(C_{\text{ref}}+C_{p})\omega -L_{S}^{\prime }\omega {\left(C_{\text{ref}}\omega \right)}^{2}≃C_{eq}\omega \Delta V_{C} \end{array}$$

Likewise, with reference resistors *Y* ≡ *G_ref_* = 1/*R_ref_*:

(18) $$\{\begin{array}{l} -G_{p}-G_{\text{ref}}+G_{\text{ref}}^{2}r_{S}≃G_{eq}\Delta V_{G}\\ -(C_{p}+0.10)\omega +G_{\text{ref}}\varphi_{G/C}+L_{S}^{\prime }\omega G_{\text{ref}}^{2}≃C_{eq}\omega \Delta V_{C} \end{array}$$

The value 0.10 pF added to *C_p_* in the last equation takes into account the stray capacitance of *R_ref_*.

Capacitance *C_p_* in Equation (18) includes the contributions from the air layer around electrodes and from the attachment of reference components to electrodes. Both are estimated and subtracted from *C_p_* to obtain the actual value of *C_p_* necessary to treat the measures of the sensor when inserted in the medium under study.

**Figure 7.**
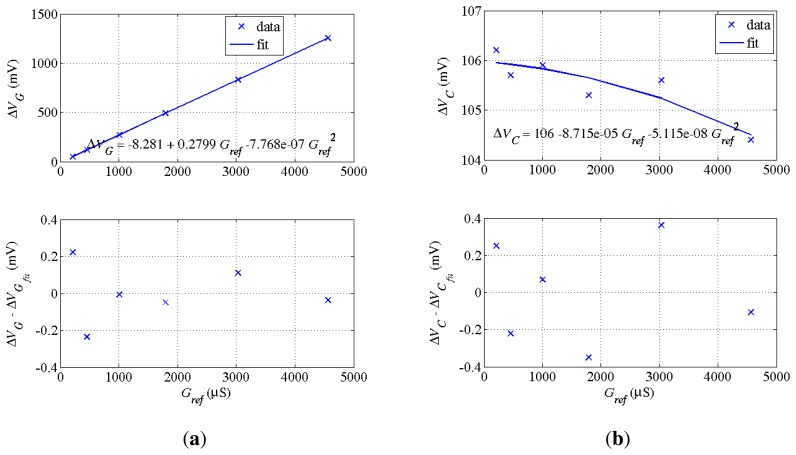
Sensor output Δ*V_G_* (**a**) and Δ*V_C_* (**b**) as a function of the reference *G_ref_* = 1/*R_ref_* using calibration data and Equation (19). For each voltage, the difference in mV between data and its fit from Equation (19) are also plotted. Channel parameters are deduced from the fits. (**a**) Plot Δ*V_G_* (*G_ref_*) and difference with fit. Hence, *G_eq_* = −3.57 *μ*S·mV^−1^. *r_S_*= 2.8 Ω; (**b**) Plot Δ*V_C_* (*G_ref_*) and difference with fit. Hence, *φ_G/C_* = 0.3110^−3^ rad.

For each channel, the adjustment of calibration data with Equations (17) and (18) yields the eight coefficients of a channel, as shown in Figure 7a for Δ*V_G_* and Δ*V_C_* as a function of *G_ref_* = 1/*R_ref_*.

### 3.2. Conversion of Sensor Output into Admittance

Once the coefficients are obtained from calibration, raw sensor outputs are converted into *Y* using first Equation (13) and then Equation (14). Inversion of Equation (15) is also required:

(19) $$Y=-Y_{p}+\frac{Y_{x}}{1-Z_{S}Y_{x}},$$

Eventual verification and/or adjustment (usually slight) of channel parameters determined from approximate Equations (17) and (18) are achieved using the more accurate Equations (14) and (19). *G* or *C* deduced from the latter equations are compared with the actual values of the reference admittances.

### 3.3. Uncertainties

From the repeatability of measurements, uncertainty on sensor raw output $\Delta V_{Y}^{\prime }$ (with *Y* corresponding to either *G* or *C*) is estimated as (expressed in standard deviation):

(20) $$\delta \Delta V_{Y}^{\prime }=+/-0.5+/-0.5\%\Delta V_{Y}^{\prime }(\text{mV})\;\text{mV}$$

The bridge output resolution is lower than 0.1 mV. Accuracy takes into account the procedure of digital acquisition (average over at least 50 points sampled at a frequency of 5 kHz).

#### 3.3.1. Uncertainties Due to Diaphony

However, further data treatments increase uncertainties. A major one results from the diaphony correction. The standard deviation of diaphony coefficients to fit calibration data according to the procedure in Section 3.1.2 is about 2%, *i.e*, *δay_km_* = +/−2%*ay_km_*. At worst, from the values given in Section 2.2.2, *δay_km_* ∼ 4 10^−4^ and *δf_km_* ∼ 2 10^−7^ mV^−1^.

Relations between standard deviations of the quantities given in Equation (13) are derived from the equation itself using the stochastic theory with normal distributions. Working with a generic form where *Y* corresponds to either *G* or *C* while *X* is its counterpart, either *C* or *G*, we have:

(21) $$\begin{array}{ll} \delta \Delta V_{Yk}^{2} & ≃\delta \Delta V_{Yk}^{\prime 2}+\sum_{m\neq k}[\delta {\left\{ay_{km}\Delta V_{Ym}^{\prime }\right\}}^{2}\\ & +\delta {\left\{f_{km}\Delta V_{Ck}^{\prime }\Delta V_{Ym}^{\prime }\right\}}^{2}+\delta {\left\{f_{km}\Delta V_{Gk}^{\prime }\Delta V_{Xm}^{\prime }\right\}}^{2}] \end{array}$$

Taking into account only the main contributions using the above values for *δay_km_* and *δf_km_*:

(22) $$\begin{array}{ll} \delta \Delta V_{Yk}^{2} & ≃\delta \Delta V_{Yk}^{\prime 2}+\sum_{m\neq k}[\delta ay_{km}^{2}\Delta_{Ym}^{\prime 2}\\ & +\delta f_{km}^{2}{\left(\Delta V_{Ck}^{\prime }\Delta V_{Ym}^{\prime }\right)}^{2}+\delta f_{km}^{2}{\left(\Delta V_{Gk}^{\prime }\Delta V_{Xm}^{\prime }\right)}^{2}] \end{array}$$

From Equation (22), we consider two cases, one with small permittivity and conductivity contrasts between channels and another one more critical, corresponding to higher contrasts adverse to channel *k*.

**The Case of Small Contrasts** The situation is modeled by $\Delta V_{Ym}^{\prime }\sim \Delta V_{Yk}^{\prime }$ and $\Delta V_{Xm}^{\prime }\sim \Delta V_{Xk}^{\prime }$. The term $\delta ay_{km}\Delta V_{Ym}^{\prime }$ is of order $2\;10^{-4}\Delta V_{Yk}^{\prime }$, which is negligible relative to $\delta \Delta V_{Yk}^{\prime }$ (Equation (20)). The two last terms in the right-hand side of Equation (22) depend on the respective amplitudes of $\Delta V_{Gk}^{\prime }$ and $\Delta V_{Ck}^{\prime }$. Because conduction depends on water to form the main charger carriers, ions, it is expected that $\Delta V_{Gk}^{\prime }<\Delta V_{Ck}^{\prime }$. For instance, the lowest admittance is obtained for a dry insulating medium for which *ε_r_* is on the order of two and *G* about a few *μ*S. The output $\Delta V_{Ck}^{\prime }$ is then close to 100 mV (at 20 MHz), while $\Delta V_{Gk}^{\prime }$ is less than 10 mV. Consequently, the two terms are only meaningful for *Yk* ≡ *Gk*. They correspond approximately to $2\delta f_{km}^{2}\left(\Delta V_{Gk}^{\prime 2}\Delta V_{Ck}^{\prime 2}\right)$. At most $\delta f_{km}\left(\Delta V_{Gk}^{\prime }\Delta V_{Ck}^{\prime }\right)$ amount to $4\;10^{-4}\Delta V_{Gk}^{\prime }$ for $\Delta V_{Ck}^{\prime }\sim 2000\;\text{mV}$, which is still negligible.
**The Case of Higher Contrasts** Because of vertical adjustments in the case of too strong of an unbalance, it is realistically modeled by $\Delta V_{Ym}^{\prime }\sim 10\Delta V_{Yk}^{\prime }$ and $\Delta V_{Xm}^{\prime }\sim 10\Delta V_{Xk}^{\prime }$ with $\Delta V_{Yk}^{\prime }$ and $\Delta V_{Xk}^{\prime }\leq 200\;mV$. The term $\delta ay_{km}\Delta V_{Ym}^{\prime }$ is of order $2\;10^{-3}\Delta V_{Yk}^{\prime }$, which makes it at most comparable to $\delta \Delta V_{Yk}^{\prime }$. For *Yk* ≡ *Ck*, the two last terms in the right-hand side of Equation (22) are negligible, even with $\Delta V_{Ck}^{\prime }=200\;\text{mV}$. Like previously, the terms may be important for *Yk* ≡ *Gk*. They amount then to about $8\;10^{-12}\left(\Delta V_{Gk}^{\prime 2}\Delta V_{Ck}^{\prime 2}\right)$. At most, for $\Delta V_{Ck}^{\prime }\sim 200\text{mV}$, it is of order $32\;10^{-8}\Delta V_{Gk}^{\prime 2}$, which is still negligible.
**Overall Uncertainty** Because the uncertainty resulting from diaphony correction is at worst of the same order of that of $\Delta V_{Yk}^{\prime }$ (Equation (20)), uncertainty on Δ*V_Y_* is thus expected to be of order or lower than δΔ*V_Y_* ∼ +/−1 +/−1% Δ*V_Y_*(mV) mV.

#### 3.3.2. Uncertainties Due to Bridge Drifts

Admittance *Y_x_* is obtained from corrected output Δ*V_Y_* and Equation (14). Uncertainty is thus expressed as:

(23) $$\{\begin{array}{l} \delta G_{x}^{2}=\delta {\left\{G_{eq}\Delta V_{G}\right\}}^{2}+\delta {\left\{C_{eq}\omega \Delta V_{C}\varphi_{C/G}\right\}}^{2}\\ \delta C_{x}\omega^{2}=\delta {\left\{C_{eq}\omega \Delta V_{C}\right\}}^{2}+\delta {\left\{C_{eq}\Delta V_{G}\varphi_{G/C}\right\}}^{2} \end{array}$$

By construction, we have *C_eq_ω* ≃ *G_eq_* ≃ 3.0 *μ*S·mV^−1^. Using the generic form as in the previous paragraph:

(24) $$\delta Y_{x}^{2}=\delta {\left\{Y_{eq}\Delta V_{Y}\right\}}^{2}+\delta {\left\{X_{eq}\Delta V_{X}\varphi_{X/Y}\right\}}^{2}$$

The uncertainty of calibration on *Y_eq_* is *δY_eq_* = +/− 0.5% *Y_eq_*. Moreover, the on-board reference resistor and capacitor are measured along with *Y_x_* to detect any drift on *Y_eq_*.

Uncertainty on phase drifts *φ_X/Y_* amounts to *δφ_X/Y_* = +/−0.001 rad (see, for example, Figure 7b). Values for *φX*/*Y* are 0.01 rad at most after adjustments.

Keeping only the main contributions, Equation (25) becomes:

(25) $$\delta Y_{x}^{2}∼Y_{eq}^{2}\left\{\delta \Delta V_{Y}^{2}+\Delta V_{X}^{2}\delta \varphi_{X/Y}^{2}\right\}$$

When Δ*V_Y_* ∼ Δ*V_X_*, the relative uncertainty on *Y_x_* is dominated by that on Δ*V_Y_*. In the case of a very large voltage Δ*V_X_*, 2000 mV at most, the term Δ*V_X_δφ_X/Y_* is lower than 2 mV. Figure 7b confirms the small contribution of this term in the uncertainty when it is well taken into account.

From the uncertainty on Δ*V_Y_*, *δY_x_* should be lower than *δY_x_* ∼ +/− 6 +/−1% *Y_x_*(*μ*S) *μ*S.

#### 3.3.3. Uncertainties Due to Channel Parasitic Admittances

The admittance *Y* under study is deduced from Equation (19). Hence, its uncertainty is:

(26) $$\delta Y^{2}≃\delta Y_{p}^{2}+\delta Y_{x}^{2}+\delta Z_{S}^{2}Y_{x}^{4}+4Z_{S}^{2}Y_{x}^{2}\delta Y_{x}^{2},$$

As *Z_S_* is about a few ohms and *Y_x_* is lower than 10 mS, the product *Z_S_Y_x_* is smaller than 0.02. The last term in Equation (26) is thus negligible relative to $\delta Y_{x}^{2}$

With *δZ_S_* about +/− 0.1 Ω, the term $\delta Z_{S}Y_{x}^{2}$ is lower than +/−10^−3^ *Y_x_μ*S, which makes it negligible.

In summary, uncertainty on *Y* is dominated by that on *Y_x_*, *i.e.*, *δY* ∼ +/−6+/−1%*Y*(*μ*S) *μ*S at worst. From Equation (7), the uncertainty on permittivity is *δε_r_* ∼ +/−0.06+/−2%*ε_r_* for *ε_r_*, and *δσ* ∼ +/−0.5+/− 1%*σ*(*μ*S·m^−1^) *μ*S·m^−1^ for *σ*.

## 4. Autonomous Sensor with a Radio Link: Other Features

Sections 2 and 3 have described the sensor layout and measurement technique, the physical equations to convert the sensor output into admittance or complex permittivity and the uncertainty on the final result.

The last step towards the most demanding applications in continuous and *in situ* monitoring is the addition of the capabilities of autonomy and wireless communication.

To make the sensor autonomous in data acquisition, bridge output is digitalized, pretreated and stored owing to a micro-controller (32-bit ARM Cortex M0 Micro-controller 64 KB flash 8 KB SRAM). Its analog to digital converters have a dynamic of 16 bits over −4096–4096 mV range. Its logical outputs operate the relays of the bridge via opto-couplers. Memory can accommodate 20,000 points (which also include the data for temperature and outputs of a second bridge; see below). This corresponds to 28 days of trial with measures carried out every two minutes, the lowest period.

Stored data are regularly sent to a distant station (a laptop equipped with a radio module). Radio communication follows a proprietary protocol developed by the companies, Full Electronic System (Eybens, France) and Bleu Solid (Pomponne, France). Digital information is transferred using a frequency-shift keying modulation scheme. The frequency carrier is 869.525 MHz; the transfer rate is 4.8 kbit·s^−1^, and the maximal range is 1–2 km (for a 500-mW powered antennae at 3-m height depending on weather and obstacles). The system is able to operate 48 independent sensors (for successive connections to all sensors in less than two minutes).

A micro-controller and integrated circuits for radio communication are mounted on a printed board closely connected to the bridge board. Both are placed inside the polyvinyl chloride tube above the discontinuous cylinder (see Figure 2b).

The sensor is made autonomous in energy owing to a pack of seven rechargeable NiMH cells located inside the other aerial tube (see Figure 2). Other choices to extend the energy capacity are envisioned, such as rechargeable Li ion batteries. The energy management of the sensor requires one to activate the boards for less than a few seconds at each measure. The power requirement of one bridge board amounts to 4 W. Its integrated circuits need a minimal time of 2 s to warm up before measuring.

Calibration is carried out using the same chain of measurement and with the same conditions as during *in situ* monitoring in order to include any effects of the settings on the calibration coefficients.

As the interpretation of data of medium permittivity and conductivity can require the local temperature of the medium [15], each sensor channel disposes of a thermometer. Temperature differences between channels can also provide information on the water vertical flow of the medium or the soil temperature gradient due to heat exchanges at the soil surface. Each thermometer is located within the Delrin frame of the discontinuous cylinder in thermal contact, yet not the electrical one, with the steel tube. The thermometer manufactured by the company, Innovative Sensor Technology (Ebnat-Kappel, Switzerland), consists of a thin-film of high purity platinum deposited on a ceramic substrate of 5 mm in length, 2.5 mm in width and 1.3 mm thick. The Pt film is laser trimmed to produce a *R*_0_ = 1000.0 Ω resistance at 0 °C. Its temperature coefficient is *α* = 1/*R*_0_*dR*/*dT* = 0.00385 K^−1^. Two Cu/Ag 30–40 cm-long wires connect each Pt thermometer to a 16-bit analog-to-digital converter in the acquisition and communication board through a Wheatstone bridge. The converter covers a temperature in the range from −30 to 50 °C with a theoretical resolution of 1.2 mK. We have calibrated each thermometer to obtain an uncertainty as low as *δT* = +/− 0.03 +/− 0.05% *T* (°C).

To reduce the costs of one sensor, all of its electronic operations, either analog or digital, are performed by integrated circuits and components mounted on two or three printed boards. The choice of materials—steel tubes and plastics—the mechanical workings and the number of distinct pieces are kept as simple or as low as possible, given the requirements of water tightness and electrode resilience over time, as well as the constraints of additional measurements, such as temperature.

**Figure 8.**
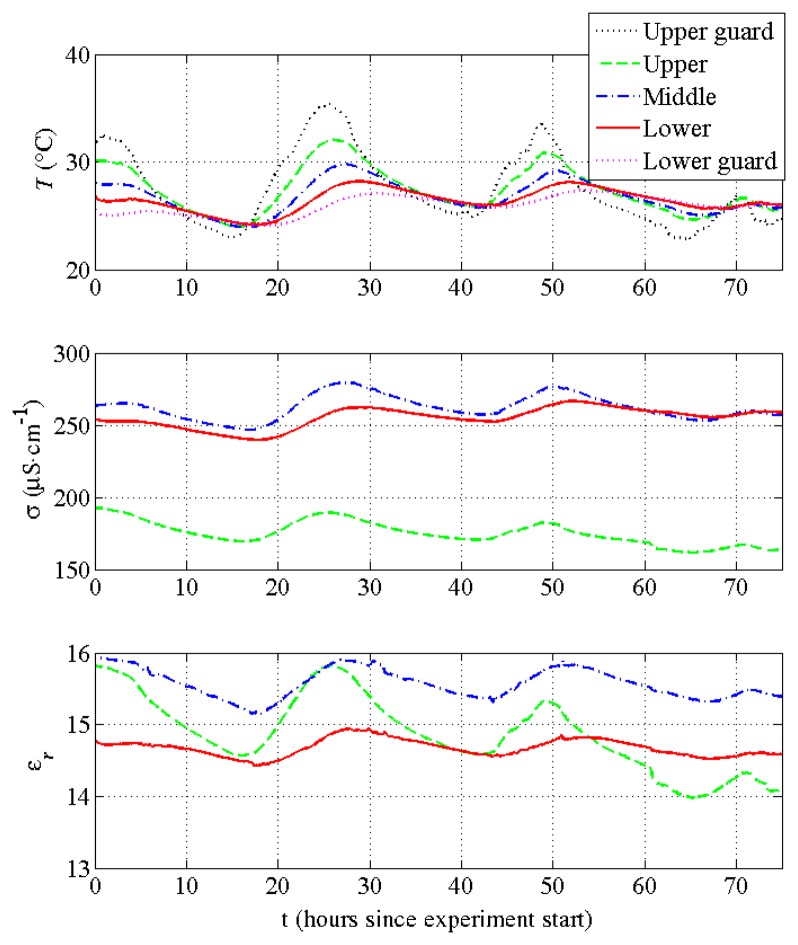
Four-day trial started at 13:54 GMT, 17 July 2014. Data of temperature for all five channels, permittivity and conductivity at 20 MHz for the three middle channels (see Figure 1). They have been recorded with a five-minute resolution. Data accuracy is specified in the text.

Figure 8 shows sensor data during a trial in a soil. The variations of temperature, permittivity and conductivity with time and depth seem consistent with what is expected for a sand with some amount of clay. As sensor improvements are still ongoing, we do not consider these data as rigorous measurements for an in-depth analysis of soil moisture and salinity.

The micro-controller is also able to drive a second bridge on a separate board closely connected to the first bridge and micro-controller boards (Figure 2b). The bridge operates at a lower frequency, such as 1 and 10 MHz, in order to derive supplementary information on porous medium by comparison with the data of the first bridge taken quasi-simultaneously (less than 100 ms).

Less sophisticated sensors, such as one simply made of two metallic rods, can be derived from the present one.

## 5. Conclusions

The article has described an admittance meter to estimate accurately at different depths the apparent relative permittivity *ε_r_* and conductivity *σ*, as well as the temperature of a porous medium, such as a soil. It will thus provide reliable inputs to a dielectric model of the medium in order to derive some of its characteristics, such as its humidity and conductivity.

The sensor is made of five stacked capacitors or channels along two stainless steel cylinders. Metallic tubes constitute the capacitor electrodes, which are inserted into the medium. Their size, each 50 mm high and long, with a distance of 100 mm, permit one to scan a significant volume by capacitor, typically 1 L, to provide the intrinsic average permittivity of the medium.

The measurement technique relies on a self-balanced bridge operating at a frequency *f* in the range 1–20 MHz. It provides two constant voltages related respectively to the conductance *G* and capacitance *C* of the admittance *Y* of each sensor capacitor.

The large size and high operating frequency enhance the electromagnetic effects distorting the bridge outputs, such as the leads' self and mutual inductances, as well as bridge imperfections. Both electric circuit theory and thorough calibration procedures permit one to take into account all of these effects with the physical parameters. The resulting uncertainty on *Y* is of order *δY* ∼ +/− 6 +/− 1% *Y*(*μ*S) *μ*S. Owing to the use of guards to prevent the field fringing effect, admittance is easily converted into medium permittivity. Uncertainty on permittivity is then *δε_r_* ∼ +/− 0.06+/− 2%*ε_r_* for *ε_r_*, and *δa* ∼ +/− 0.5 +/− 1% *σ*(*μS* · m^−1^) *μ*S ·m^−1^ for *σ*.

The preliminary results during a four-day trial are presented.

To better characterize the dependence of medium permittivity on frequency, the sensor is also able to retrieve it at two distinct frequencies using two bridges. Thanks to theoretical insight on parasitic effects on bridge output, it is possible to extend *f* to 30 MHz.

Less sophisticated sensors, such as one simply made of two metallic rods, can be derived from the present one.

We have equally described developments to make the sensor autonomous on digital acquisition, basic data treatment and storage, as well as able to transfer data by a radio link. These steps are prerequisites for a wireless network of sensors to meet the most demanding applications, especially to study soil moisture evolution and distribution over a catchment basin.
